# Comparison between Abbott m2000 RealTime and Alinity m STI systems for detection of *Chlamydia trachomatis*, *Neisseria gonorrhoeae*, and *Mycoplasma genitalium*

**DOI:** 10.1007/s10096-020-04135-9

**Published:** 2021-03-15

**Authors:** Björn Herrmann, Karin Malm

**Affiliations:** 1 Department of Clinical Microbiology, Uppsala University Hospital, SE-751 85 Uppsala, Sweden; 2 Section of Clinical Bacteriology, Department of Medical Sciences, Uppsala University, Uppsala, Sweden

**Keywords:** *Chlamydia trachomatis*, *Neisseria gonorrhoeae*, *Mycoplasma genitalium*, Nucleic acid amplification tests

## Abstract

The new Abbott Alinity m STI Assay was compared with Abbott m2000 RealTime PCR. For *Chlamydia trachomatis*, 26 (7.5%) of 347 samples were positive in the Alinity assay and 24 (6.9%) in the m2000 assay. Corresponding figures for *Neisseria gonorrhoeae* were 23 (6.6%) and 17 (4.9%). For *Mycoplasma genitalium*, 22 (7.9%) of 279 samples were positive in the Alinity assay and 18 (6.5%) in the m2000 assay, for which DNA extraction was performed on an m2000sp instrument combined with in-house real-time PCR. The Alinity assay has at least the same sensitivity as the m2000 assay. The specificity was evaluated by discrepancy analysis.

**Supplementary Information:**

The online version contains supplementary material available at 10.1007/s10096-020-04135-9.

Over the last three decades, continuous advances have been made in the molecular diagnostics of bacteria causing sexually transmitted infections. Diagnostic accuracy has improved, automation has increased, and multiplex detection of target organisms is now common. In the last two decades, detection of *Chlamydia trachomatis* (CT) and *Neisseria gonorrhoeae* (NG) has typically been performed as a duplex test in the major commercial assays, with *Mycoplasma genitalium* (MG) now added to such assays. Although not relevant for all countries and settings, the parasite *Trichomonas vaginalis* is also included.

The Abbott Alinity platform is a new random-access system developed to replace the m2000 RealTime platform. In this study, the Alinity m STI Assay was compared with the Abbott m2000 RealTime System for detection of CT/NG, and for MG with DNA extraction on Abbott m2000sp (as for CT/NG) and detection by an adapted in-house real-time PCR [1]. In the in-house real-time PCR, 10 μL DNA extract from the m2000 procedure was used. This combination of DNA extraction on m2000 and in-house PCR detection is referred to here as “m2000 assay” for MG. Samples were also analyzed for *Trichomonas vaginalis* in the Alinity assay.

Duplicate samples from urine (153), cervix/vagina (130), rectum (59), or throat (5) were taken from persons attending an STI clinic for suspected CT/NG/MG infection. Abbott multi-Collect Specimen Collection Kits were used for the m2000 RealTime System and Alinity-m multi-Collect Specimen Collection Kits for the Alinity-m STI. Samples were taken from consecutive patients and the sample order for the two collection kits was shifted every week to avoid bias.

In our laboratory, Alinity NG-positive samples are always tested by a confirmatory duplex in-house PCR using *opa* and *porA* as target genes [2]. Since in the Alinity system the DNA eluate is used directly for detection, with no leftover volume for additional tests, a separate DNA extraction had to be used for confirmatory PCR. This was done using a 200 μL sample diluted in 200 μL nuclease-free water for DNA extraction in a magLEAD-12gC extraction robot (Precision System Science, Japan). In the confirmatory PCR, 5 μL eluate was analyzed.

Evaluation of NG and MG detection was performed on all Alinity-positive samples over a 6-month period, including clinical data and antibiotic treatment. From all Alinity MG–positive samples, DNA was extracted with magLEAD and retested with PCR (Diagenode S-DiaMGRes-kit, Diagenode Diagnostics**,** Liège, Belgium).

The performance of the Alinity-m STI system was also tested on proficiency panels. For CT/NG, the QCMD 2020 DNA EQA panels QAB004101_2 and QAB034126_2 https://qcmd.org were used, and for MG panel 2019-288 from the quality assessment organization EQUALIS https://www.equalis.se/en/about-us/.

The data used in this study were part of routine practices in the hospital prior to and after implementation of a new method (see supplement). Data on antimicrobial treatment were taken from case records. The study was approved by the Regional Ethical Review Board in Uppsala, Sweden (ref. 2020-04572).

Analysis of CT/NG was performed on 347 samples. For CT, 26 samples (7.5%) were positive in the Alinity assay and 24 (6.9%) in the m2000 assay. Corresponding figures for NG were 23 (6.6%) and 17 (4.9%); see Table 1. The number of detected cases was not statistically different for either CT or NG when the two assays were compared. Using the m2000 assay as reference, the Alinity assay for CT detection had a sensitivity of 95.8% (78.9–99.9) and a specificity of 99.1% (97.3–99.8). For NG the corresponding figures were 100% (80.5–100.0) and 98.2% (96.1–99.3). Agreement for the two assays was 98.8% (97.1-99.7, Table 1) for CT and 98.3% (97.1-99.7) for NG. The distribution of samples by different types is shown in Table 2.

**Table 1:** Analysis of 347 duplicate samples with suspected CT/NG-infection collected in Abbott multi-collect m2000 tubes and in Alinity m STI tubes. Analyzed with Abbott m2000 and Alinity m

	*C. trachomatis*	*N. gonorrhoeae*	*M. genitalium*
	No. of samples	No. of samples	No. of samples
m2000 positive/Alinity m positive	23	17	17
m2000 negative/Alinity m positive	3	6	5
m2000 positive/Alinity m negative	1	0	1
m2000 negative/Alinity m negative	320	324	256
	347	347	279
Agreement % Kappa value	98.8 (97.1–99.7) 0.914 (0.830–0.998)	98.3 (96.3–99.4) 0.841 (0.717–0.966)	97.8 (95.4–99.2) 0.839 (0.712–0.965)

**Table 2:** Distribution of sample types analyzed with Alinity m STI^a^

		*C. trachomatis*		*N. gonorrhoeae*		*M. genitalium*		
Specimen type	No. of samples	No. of positive	No. of negative	No. of positive	No. of negative	No. of samples	No. of positive	No. of negative
Urine	153	6	147	8	145	123	9	114
Throat	5	1	4	2	3	0	0	0
Cervix/vagina	130	13	117	8	122	111	12	99
Rectum	59	6	53	5	54	45	1	44
	347	26	321	23	324	279	22	257

^a^All samples also analyzed with the m2000 assay, but the low number of different positive sample types did not allow relevant statistical analysis for comparison

The six NG samples that were Alinity-positive/m2000-negative had cycle of threshold (Ct) values ranging from 22.2 to 38.9 (22.2; 24.4; 24.7; 35.6; 37.0; 38.9). By mistake these six samples were not tested for discrepancy analysis with the confirmatory duplex in-house PCR by Goire et al. [2]. However, of 67 Alinity NG–positive samples over 6 months in our laboratory, 51 were unequivocally confirmed in the duplex PCR, with detection of both targets. Of the remaining 16 samples, four were interpreted as representing true NG infection, one of which was negative for the *porA* target; see supplement. Nine samples were determined as NG trace DNA after antibiotic treatment, with a mean Ct value of 36.7 in Alinity (range 35.1–39.5), and three as an unspecific reaction, with a mean Ct value of 36.4 in Alinity (range 33.9–38.9). Thus, Alinity-positive samples that could not be confirmed by in-house PCR indicate low copy numbers of the target, leading to discrepant results.

Analysis of MG was performed on 279 samples (a subset of the 347 samples), of which 22 (7.9%) were positive in the Alinity assay and 18 (6.5%) in the m2000 assay. The five samples that were Alinity-positive/m2000-negative had cycle numbers in the range 35.7–39.8, probably indicating higher sensitivity for Alinity. Agreement for the two assays was 97.8% (95.4–99.2).

Over 6 months, 167 Alinity MG–positive samples were retested with an additional PCR (Diagenode) and 159 were positive in both assays. The remaining eight samples had a mean Ct value of 34.7 (31.9–37.5) in Alinity and were negative in the Diagenode assay.

In 347 samples, *T. vaginalis* was not detected in the Alinity assay. This was expected, since the prevalence of this agent is almost zero in Sweden and detection is confined to sporadic imported cases [3].

In the external quality assessment panels of five samples for CT and NG, the Alinity assay had expected detection results for both bacteria in all samples, with Ct values between 24.9 and 35.8 (Supplementary table 1). Analysis of the MG panel produced a similar outcome: all six samples gave expected results in the Alinity assay, with a Ct value range of 30.5–38.2. A point to be noted was that the weakest positive sample in the Alinity assay was negative when the same panel was tested in the m2000 assay. Of the 33 participating laboratories, 18% did not detect MG in that sample.

This comparative study was undertaken with the objective of replacing the m2000 platform with the next-generation platform Alinity. One strength of the study is that it was performed in a clinical laboratory without additional precautions beyond those routinely applied, i.e., the outcome reflects what is achieved in a real-world laboratory setting. The study design also had limitations. First, the number of tests did not achieve sufficient statistical power to detect any differences in sensitivity between the Alinity and m2000 assays for CT/NG detection. Nevertheless, our results indicate that the Alinity-m STI assay had at least the same sensitivity as the m2000 assay for CT/NG detection. Since MG is not included in the Abbott m2000 assay, comparison between the Alinity assay and a combination of DNA extraction on m2000 and an in-house PCR method is not straightforward. But the outcome for clinical samples and the proficiency panel indicates that the Alinity assay has sufficient detection capacity to be used. Since the copy number of MG is up to a hundred times lower in clinical samples compared to CT [4], test sensitivity is essential for detection of MG.

Another limitation of our study is that the number of specific sample types did not permit separate analysis for urine, cervical/vaginal, rectal, and throat specimens. According to the package insert for the Alinity-m STI AMP Kit (version 53-608074/R1), the test is only validated for samples from the cervix/vagina and urine for CT/NG and only for endocervical specimens from for MG. Table 2 indicates that detection is entirely possible from all specimen types, although the throat is rarely a clinically relevant target for MG [5].

A technical limitation of the automated Alinity system with integrated DNA extraction and target detection is that it does not permit downstream nucleic acid amplification tests. Confirmation of NG, which is needed in low-prevalence settings [6], and antibiotic resistance detection for MG therefore require separate DNA extraction from the Alinity-m multi-collect specimen sample tube (09N19-001). This sample tube contains different components, including lysing agents. Efficient DNA extraction in other systems is consequently often suboptimal, since the compounds from different systems are mixed and disturb the balance of components. To avoid clogging, in our laboratory we dilute the sample from the Alinity tube twofold in water before DNA extraction in the magLEAD-12gC extraction robot.

Evaluation of nucleic acid amplification tests for CT/NG has previously been controversial, and their performance has been questioned even after careful discrepancy testing [7, 8]. On the other hand, well-established commercial test systems provide high enough sensitivity for what is clinically relevant and may even be so sensitive that contamination of samples may lead to false-positive results [9–11]. Specificity has been a major problem for some amplification tests regarding NG, but the newest generation of test systems have acceptable performance [12], although not in low-prevalence settings where confirmation tests are needed to avoid false-positive results [13].

In summary, the Alinity-m STI system provides a user-friendly automatic system with adequate detection of CT, NG, and MG.

## Supplementary Information

ESM 1 (DOCX 24 kb)
