# Supplementary material for: Comparison between Abbott m2000 RealTime and Alinity m STI systems for detection of Chlamydia trachomatis, Neisseria gonorrhoeae, and Mycoplasma genitalium
Source: Eur J Clin Microbiol Infect Dis. 2021 Mar 15;40(10):2217–20. doi: 10.1007/s10096-020-04135-9 (PMC8449771; doi:10.1007/s10096-020-04135-9)
Supplement: Supplementary file 1 — (DOCX 24 kb) [file 10096_2020_4135_MOESM1_ESM.docx]

European Journal of Clinical Microbiology & Infectious Diseases
Herrmann and Malm, Comparison between Abbott m2000 RealTime and Alinity m STI systems for detection of Chlamydia trachomatis, Neisseria gonorrhoeae and Mycoplasma genitalium

**Supplementary information**

**Description of samples used in the study**

Comparison between Abbott Alinity m STI and Abbott m2000 RealTime-PCR for Chlamydia trachomatis (CT) and Neisseria gonorrhoeae (NG):
347 samples (153 urine; 130 cevix/vagina; 59 rectum; 5 throat) from patients attending an STI clinic between 7 October 2019 – 13 January 2020.

279 of the 347 samples were also tested for Mycoplasma genitalium (MG) on Alinity m STI and in the m2000, for which DNA extraction was performed on an m2000sp instrument combined with in-house real-time PCR [**1].**

The 347 samples were also tested for Trichomonas vaginalis by the Alinity m STI assay.

Additional testing in routine diagnostics over a six month period

67 initially NG-positive samples in Alinity m STI were also retested by a confirmatory duplex PCR **using opa and porA as target genes [2].** DNA was first extracted from the Alinity m multi-Collect Specimen Collection Kits with magLEAD 12gC extraction robot (Precision System Science, Japan)

167 initially MG-positive samples in Alinity m STI were also tested by a commercial PCR for detection of MG and macrolide resistance. DNA was first extracted from the Alinity m multi-Collect Specimen Collection Kits with magLEAD 12gC extraction robot and retested with PCR (Diagenode S-DiaMGRes-kit, Diagenode Diagnostics, Liège, Belgium).

External quality assessment panels

For CT (5 samples) QCMD 2020 DNA EQA panel QAB004101_2 and for NG (5 samples) panel QAB034126_2 <https://qcmd.org> were used.

For MG (6 samples) panel 2019-288 from the quality assessment organization EQUALIS <https://www.equalis.se/en/about-us/> was used.

References

1. Jensen JS, Björnelius E, Dohn B, Lidbrink P (2004) Use of TaqMan 5' nuclease real-time PCR for quantitative detection of *Mycoplasma genitalium* DNA in males with and without urethritis who were attendees at a sexually transmitted disease clinic. J Clin Microbiol 2004, 42:683-692.

2. Goire N, Nissen MD, LeCornec GM, Sloots TP, Whiley DM. A duplex *Neisseria gonorrhoeae* real-time polymerase chain reaction assay targeting the gonococcal porA pseudogene and multicopy opa genes. Diagn Microbiol Infect Dis 2008, 61:6-12.

**Statistics**

Statisticial calculations of sensitivity, specificity, agreement and kappa values with confidence intervals were performed on the software R version 4.0.3 (2020-10-10), x86_64-w64-mingw32.

**Supplementary table 1. Discrepancy analysis of *Neisseria gonorrhoeae*-positive samples in the Alinity m STI assay and the outcome in confirmation tests by duplex in house-PCR**

| **Date** | **Sample no.** | **Sample site** | **Alinity Ct-value^a^** | **Duplex  PCR** | ***opa*  target**  **result** | ***opa* Ct-value^a^** | ***porA*  target** | ***porA***  **Ct-value^a^** | **Interpretation** | **Comment** |
| --- | --- | --- | --- | --- | --- | --- | --- | --- | --- | --- |
| 12/2 | 3231 | Vagina | 36.2 | + | + | 36.6 | - | 0 | NG trace DNA | Sample taken 14 days after ceftriaxone treatment |
| 18/2 | 3614 | Throat | 39.5 | - | - | 0 | - | 0 | NG trace DNA | Samples taken the same day were confirmed PCR positive in urine and urethra culture positive. Ceftriaxone treatment the same day. Mecillinam treatment for suspected urinary tract infection initiated 17 days earlier. |
| 20/2 | 3860 | Urine | 38.2 | - | - | 0 | - | 0 | NG trace DNA | Ceftriaxone treatment given 8 days earlier.  Samples taken 14 days earlier were PCR positive in urine and culture positive in urine. |
| 24/2 | 4009 | Throat | 28.9 | + | + | 27.4 | - ^b^ | 0 | NG infection | Ceftriaxone treatment given 13 days after sampling. Culture negative 13 days later and PCR negative 36 days later. |
| 26/2 | 4510 | Urine | 38.0 | - | - | 0 | - | 0 | NG trace DNA | Sample taken 15 days after ceftriaxone treatment when the patient also was NG-positive in urethra culture. |
| 28/2 | 4928 | Throat | 35.9 | - | - | 0 | - | 0 | NG trace DNA | Sample taken 60 days earlier with confirmed PCR-positive in throat. Patient delayed ceftriaxone treatment until 20 days before sample 4928 was taken. |
| 24/3 | 6907 | Throat | 36.1 | - | - | 0 | - | 0 | NG trace DNA | Sample taken 7 days after previous confirmed PCR-positive throat sample and 23 days after ceftriaxone treatment |
| 13/5 | 9479 | Throat | 37.6 | - | - | 0 | - | 0 | NG infection | Urine sample taken the same day was confirmed PCR-positive. Ceftriaxon treatment given 6 days after sampling. |
| 15/5 | 9882 | Throat | 37.7 | - | - | 0 | - | 0 | NG infection | The same day urine sample was confirmed PCR-positive and ceftriaxone treatment was given.  Previous NG-positive sample tested about one week earlier in another laboratory. |
| 15/5 | 9887 | Rectum | 38.9 | - | - | 0 | - | 0 | Unspecific reaction, no NG. | Throat and urine samples taken same day was PCR-negative.  No treatment given. |
| 27/5 | 10479 | Throat | 34.4 | - | - | 0 | - | 0 | NG infection | Rectum sample taken same day was confirmed PCR-positive. Ceftriaxone treatment given 8 days later. |
| 27/5 | 10611 | Vagina | 36.5 | - | - | 0 | - | 0 | Unspecific reaction, no NG. | Follow up sample from vagina 6 days later was PCR negative and culture negative.  No treatment given. |
| 29/6 | 12569 | Throat | 33.9 | - | - | 0 | - | 0 | Unspecific reaction, no NG. | Rectum and urine samples taken the same day were PCR-negative and culture negative in throat, rectum and urine. No treatment given |
| 3/7 | 12894 | Throat | 35.1 | - | - | 0 | - | 0 | NG trace DNA | Sample taken 23 days after treatment with ciprofloxacin 500 mg x 1. Throat culture was positive 38 days earlier. |
| 5/8 | 14727 | Throat | 35.5 | - | - | 0 | - | 0 | NG trace DNA | Sample taken 17 days after ceftriaxone treatment and a culture positive in throat sample. |
| 5/8 | 14732 | Throat | 35.5 | - | - | 0 | + | 39.5 | NG trace DNA | The sample was taken 24 days after a culture positive throat sample and ceftriaxone treatment. |

^a^ Ct: amplification cycle of threshold number for target detection. Ct denoted as CN in the Alinity assay description.

^b^ The *porA* gene in *N. gonorrhoeae* is conserved but variation in the target has been noted and is likely to explain the negative result. See Ison CA, Golparian D, Saunders P, Chisholm S, Unemo M (2013) Evolution of Neisseria gonorrhoeae is a continuing challenge for molecular detection of gonorrhoea: false negative gonococcal porA mutants are spreading internationally. Sex Transm Infect 89:197-201.

+: target detected; -: target not detected; 0: no Ct-value

**Supplementary table 2. Performance of the Alinity m STI assay in analysis of two proficiency panels:
For Chlamydia trachomatis/Neisseria gonorrhoaeae the QCMD 2020 DNA EQA panels QAB004101_2
and QAB034126_2 and for Mycoplamsa genitalium panel 2019-288 from EQUALIS**

| Panel | Sample | Expected result according to panel provider | Result from local laboratory  Alinity | Cycle of threshold |
| --- | --- | --- | --- | --- |
| QAB004101_2 | CTDNA20C1-01 | Frequently detected | Positive | 34.1 |
|  | CTDNA20C1-02 | Detected | Positive | 35.8 |
|  | CTDNA20C1-03 | Frequently detected | Positive | 35.7 |
|  | CTDNA20C1-04 | Negative | Negative |  |
|  | CTDNA20C1-05 | Frequently detected | Positive | 35.3 |
| QAB034126_2 | NgDNA20C1-01 | Frequently detected | Positive | 29.9 |
|  | NgDNA20C1-02 | Frequently detected | Positive | 24.9 |
|  | NgDNA20C1-03 | Frequently detected | Positive | 27.2 |
|  | NgDNA20C1-04 | Negative | Negative |  |
|  | NgDNA20C1-05 | Frequently detected | Positive | 27.5 |
| 2019-288 | 2019:01/A | Positive (strong) | Positive | 30.5 |
|  | 2019:01/B | Positive | Positive | 34.3 |
|  | 2019:01/C | Positive | Positive | 34.0 |
|  | 2019:01/D | Negative | Negative |  |
|  | 2019:01/E | Positive (weak) | Positive | 38.2 |
|  | 2019:01/F | Positive | Positive | 33.0 |

For QCMD panels information is available at <https://qcmd.org>
For EQUALIS panels information is available at <https://www.equalis.se/en/about-us/>.
